# High mobility group A2 (HMGA2) protein tissue levels and its association with clinicopathological features in bladder cancer patients

**DOI:** 10.1007/s12672-025-03462-7

**Published:** 2025-08-25

**Authors:** Feras Alsoufi, Almoutassem Billah Zetoune

**Affiliations:** Department of Biochemistry and Microbiology, Faculty of pharmacy, Damascus University, Damascus, Syria

**Keywords:** Bladder cancer, HMGA2, IHC, Stage, Grade, NMIBC, MIBC.

## Abstract

**Background:**

Bladder cancer is the most common malignancy of the urinary tract, and is characterized by a high risk of recurrence and mortality. Therefore, the research is still ongoing to determine new molecular markers that would be a part of bladder cancer diagnosing approach. Based on the above, this study determined the level of HMGA2 protein in bladder cancer tissues for the purpose of investigating the possibility of proposing it as a promising diagnostic marker, as HMGA2 is an architectural transcription factor that regulates cell proliferation, cell differentiation, and apoptosis.

**Methods:**

Forty primary transitional cell bladder samples and 20 adjacent normal tissue samples were collected from patients at Al-Assad University Hospital in Damascus between March 2023 and January 2024. Immunohistochemical staining was used to assess HMGA2 protein levels. The relationship between HMGA2 tissue levels and clinical variables was analyzed using the Chi-Square Independence Test.

**Results:**

This study showed that HMGA2 protein was overexpressed in 70% of bladder cancer tissues, with no expression in normal tissues. Also, a High HMGA2 levels were noticed to be significantly associated with advanced tumor stage, higher tumor grade, and muscle invasion (*p* < 0.05). No significant association was found between HMGA2 levels and gender or smoking habits.

**Conclusion:**

HMGA2 protein is overexpressed in bladder cancer tissues and correlates with tumor progression and aggressiveness. HMGA2 can be considered a promising marker for bladder cancer. Further studies with larger patient cohorts are needed to confirm these findings and establish HMGA2 as a reliable diagnostic tool.

## Introduction

Bladder cancer, an occupational disease first identified among dye workers in Germany in the mid-1890s [1], is characterized by a high risk of recurrence and mortality. According to Global Cancer Statistics for 2022, there were approximately 614,298 new cases and 220,596 deaths worldwide due to bladder cancer [2]. The initial suspicion of bladder cancer often arises from the presence of hematuria, with the diagnosis confirmed through cystoscopy. A TransUrethral Resection of the Bladder Tumor (TURBT) is subsequently performed to determine the tumor’s pathological features and to assess whether the bladder’s detrusor muscle has been infiltrated [3–5]. Ongoing research is focused on identifying molecular biomarkers to enhance the diagnostic approach for bladder cancer. High Mobility Group (HMG) proteins, which are non-histone chromatin components with molecular weights ranging from 10 to 15 kDa, are of particular interest [6–8]. HMGA2, a member of the HMG family and HMGA subfamily, is characterized by distinct functional motifs that enable it to bind to specific adenine-thymine-rich regions of B-form DNA or chromatin independently of the nucleotide sequence via AT hooks ( [9, 10] [11]). This binding alters chromatin structure and regulates the expression of various genes involved in cellular processes such as proliferation, differentiation, apoptosis, and different stages of carcinogenesis [12].

HMGA2 protein levels are elevated during embryonic development but decrease or disappear in adult tissues, only to increase again during carcinogenesis [10, 11]. Studies have shown high tissue levels of HMGA2 protein and its association with clinicopathological characteristics in various cancers, including breast, liver, gastric, and bladder cancer [13–20]. This research aims to detect the frequency of HMGA2 protein presence in the bladder tissues of Syrian patients with bladder cancer across different tumor stages and grades, with the goal of suggesting its incorporation into the diagnostic approach for bladder cancer, as HMGA2 protein has been highlighted due to its prominent regulatory role in cellular signaling pathways that play a central role in carcinogenesis and tumor progression.

## Materials and methods

### Study population

Forty primary transitional cell bladder cancer samples were collected from patients in the Urology Department at The National University Hospital in Damascus between March 2023 and January 2024. Additionally, 20 adjacent normal tissue samples were surgically removed and collected from the same patients (5 cm from the tumor) and confirmed to be normal by histological examination. Despite the limited sample size, the selection criteria were rigorously defined to ensure sufficient statistical representation and result accuracy, enabling the extraction of. Valuable preliminary insights warranting further investigation.

The patient cohort consisted of 34 males and 6 females, with a mean age of 62 ± 1 years. Among these patients, 12 underwent radical cystectomy, while 28 underwent transurethral resection of bladder tumor (TURBT). All samples were obtained immediately after surgical removal, preserved in paraffin wax, and subsequently sectioned for analysis.

The general and clinicopathological features of the Participating patients are summarized in Table 1.

**Table 1:** The general and clinicopathological features of the participating patients

Parameter		Number of cases (%)
Gender	Male	34 (85%)
	Female	6 (15%)
Bladder cancer stages	pT1	29 (72.5%)
	pT2	4 (10%)
	pT3	7 (17.5%)
Bladder cancer grades	Low grade	17 (42.5%)
	High grade	23 (57.5%)
Muscle invasion status	NMIBC	27 (67.5%)
	MIBC	13 (32.5%)
Smoking habits	Non-smoker	12 (30%)
	Smoker	28 (70%)

MIBC: Muscle-Invasive Bladder Cancer, NMIBC: Non-Muscle Invasive Bladder Cancer

The previous table indicate that 85% of the patients were male and 15% were female. According to the World Health Organization (WHO) criteria and the 2002 TNM classification system, 72.5% of the patients had pT1 bladder cancer stage, 17.5% had pT3 stage, and only 10% had pT2 bladder cancer stage. Additionally, 57.5% of the patients had high-grade tumors, while 42.5% had low-grade tumors. Non-Muscle Invasive Bladder Cancer (NMIBC) was present in 67.5% of the patients, whereas 32.5% had Muscle-Invasive Bladder Cancer (MIBC). Furthermore, 70% of the patients were smokers, and 30% were non-smokers.

Patient information was obtained through personal interviews and reviewing their medical files, while clinicopathological data were sourced from the hospital pathology laboratory. This study received approval from the Biomedical Research Ethics Board (BMREC) at Damascus University (January 4, 2022) (No. 5). Tissue samples were collected from participants after they provided written informed consent.

### Inclusion and exclusion criteria

Inclusion criteria included patients with a histopathological diagnosis of transitional cell carcinoma of the bladder, newly diagnosed and untreated bladder cancer, no history of other tumors, and the availability of detailed clinical and pathological data. Exclusion criteria encompassed patients with cancers other than bladder cancer, those undergoing any type of cancer treatment, and patients highly exposed to any risk factors causing mutations.

### Immunohistochemical staining

Immunohistochemical staining was performed using an APC/CY7-linked polyclonal antibody against HMGA2 (MyBioSource, USA). Formalin-fixed, paraffin-embedded tissue Sect. (5 μm) were mounted on positively charged slides, heated at 60 °C for one hour, deparaffinized in xylol, and rehydrated with graded ethanol. Antigen retrieval was achieved by microwave boiling in EDTA buffer (pH 9) for 30 min, followed by washing, blocking endogenous peroxidase with hydrogen peroxide, and additional washing. The slides were incubated with a 1:50 diluted primary HMGA2 antibody for 45 min, linked with a secondary anti-mouse/anti-rabbit antibody for 15 min, and labeled with an HRP-conjugated Fab solution for another 15 min. Signal detection was obtained with DAB chromogen, and nuclei were counterstained with hematoxylin. Finally, the sections were dehydrated through graded alcohols, cleared in xylol, and mounted with Canada balsam for microscopic examination.

### Immunohistochemical staining evaluation

Immunohistochemical staining was evaluated by two pathologists, blinded to clinical findings and other clinicopathological data. Protein expression was assessed by scanning each whole tissue specimen at low magnification (×40) and confirmed at high magnification (×100 and ×400). The intensity of immunohistochemical staining was scored as 0 (no staining), 1 (weakly stained), 2 (moderately stained), or 3 (strongly stained). The extent of staining was scored as 0 (negative), 1 (< 10% of the tumor area stained), 2 (10–50% of the tumor area stained), or 3 (> 50% of the tumor area stained). The overall score was determined by combining the mean staining intensity and staining distribution. Scores of 0 and 1 were considered low expression, while scores equal to or greater than 2 were considered high expression as shown in Table 2.

**Table 2:** Immunohistochemical staining results of bladder tissue samples

Parameter	Score	Number of cases (%)
Staining intensity	0	4 (10%)
	1	8 (20%)
	2	20 (50%)
	3	8 (20%)
HMGA2 tissue level	Low	12 (30%)
	High	28 (70%)

### Statistical analysis

All statistical analyses were conducted using the Statistical Package for the Social Sciences (SPSS) version 25 (SPSS Inc., Chicago, IL, USA). The Chi-Square Independence Test was employed to evaluate the relationship between two qualitative variables, assessing differences in proportions. *P* < 0.05 were considered statistically significant.

## Results

### HMGA2 protein levels in bladder cancer tissue samples

**Fig. 1:**
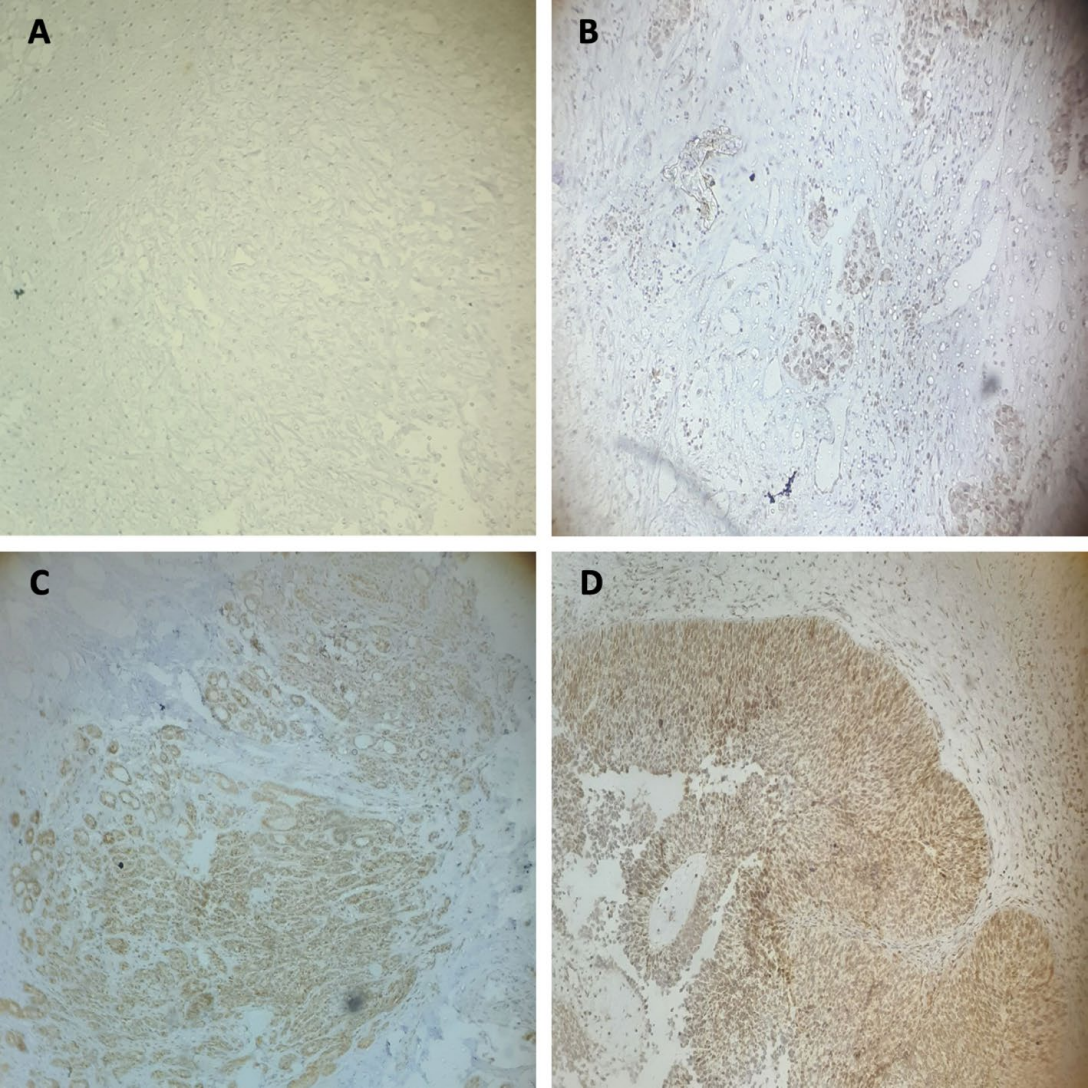
Immunohistochemical staining of HMGA2 protein in bladder cancer tissues. **A** Negative HMGA2 staining (normal bladder tissue), **B** Weak HMGA2 staining, **C** Moderate HMGA2 staining, (**D**) Strong HMGA2 staining. (**A**) and (**B**) presented low HMGA2 expression tissues, (**C**) and (**D**) presented high HMGA2 expression tissues (Magnification x10, x10)

Immunohistochemical staining revealed that 70% (28/40) of bladder cancer tissue samples exhibited high levels of HMGA2 protein, whereas no HMGA2 protein was detected in the 20 normal bladder tissue samples. In HMGA2-positive cases, brown staining was predominantly observed in the nuclei and cytoplasm of the cells as shown in Fig. 1.

### Relationship between HMGA2 tissue levels and clinical variables of bladder cancer

The immunohistochemical staining results demonstrated a statistically significant correlation between elevated HMGA2 protein levels and the progression of bladder cancer stages in the patients (*p* < 0.05). Additionally, higher HMGA2 protein levels were associated with higher bladder cancer grade (*p* < 0.05). Muscle invasion was also significantly associated with elevated HMGA2 protein levels (*p* < 0.05). However, no statistically significant relationship was found between HMGA2 protein levels and patients’ gender or smoking habits (*P* > 0.05), as shown in Table 3.

**Table 3:** The distribution of study individuals according to gender, smoking status, and clinicopathological features based on HMGA2 tissue level

Bladder Cancer Patients’ Parameters		HMGA2 tissue level		Pearson Chi-Square (P-value)
		Low Number of cases (%)	High Number of cases (%)	
Bladder cancer stages	pT1	9 (41.4%)	20 (58.6%)	0.039*
	pT2	1 (25%)	3 (75%)	
	pT3	2 (28.6%)	5 (71.4%)	
Bladder cancer grades	Low grade	8 (47.1%)	9 (52.9%)	0.043*
	High grade	9 (52.9%)	19 (82.6%)	
Muscle invasion status	NMIBC	12 (44.4%)	15 (55.6%)	0.013*
	MIBC	2 (15.4%)	11 (84.6%)	
Gender	Male	9 (26.5%)	25 (73.5%)	0.063
	Female	3 (50%)	3 (50%)	
Smoking habits	Non-smoker	6 (50%)	6 (50%)	0.071
	Smoker	6 (21.4%)	22 (78.6%)	

Values with a significant statistical difference (P-value < 0.05) were indicated by (*)

BC: Bladder Cancer, MIBC: Muscle-Invasive Bladder Cancer, NMIBC: Non-Invasive Bladder Cancer

## Discussion

HMGA2 is a protein that plays a crucial role in embryogenesis but exhibits tumorigenic properties when expressed in adult tissues [10]. Numerous studies have demonstrated that elevated levels of HMGA2 are associated with poor prognosis in various cancers [21, 22]. In the context of bladder cancer, two significant histological studies have explored the relationship between HMGA2 protein expression levels and the histological and clinical characteristics of bladder cancer, comparing these levels with those in adjacent normal tissues. The first study, conducted by Yang et. al. in China in 2011 [19], included 148 tissue samples analyzed for HMGA2 protein levels using ImmunoHistoChemical staining (IHC) and 44 tissue samples analyzed for HMGA2 mRNA levels using qRT-PCR. The second study, conducted by Ding et. al. in China in 2014 [20], included 49 tissue samples analyzed for HMGA2 protein levels using IHC. Consistent with these studies, we found that HMGA2 protein was overexpressed in bladder cancer tissues compared to adjacent normal tissues. During cancer progression, the regulatory mechanisms controlling HMGA2 gene expression are often disrupted. This disruption includes decreased methylation of the HMGA2 gene promoter and loss of miRNA-mediated regulation, which is the primary mechanism controlling HMGA2 protein expression levels. These regulatory losses are due to mutations or deletions in the 3’ UnTranslated Region (UTR) of HMGA2 mRNA [11, 23].

This study also demonstrated a positive association between increased HMGA2 protein levels and the progression of bladder cancer stage, grade, and muscle invasion, findings that align with the results of Yang et al. (2011) [19] and Ding et al. (2014) [20]. When overexpressed, HMGA2 stimulates biological pathways related to the cell cycle, apoptosis, angiogenesis, and Epithelial-Mesenchymal Transformation (EMT), which support cancer cell survival and proliferation, leading to cancer progression, increased aggressiveness, and poor prognosis [21, 22]. HMGA2 is a small, non-histone, chromatin-associated protein with no intrinsic transcriptional activity. Its AT-hook motif allows it to bind AT-rich DNA sequences with high affinity, modifying gene transcription by altering chromatin structure. This enhances or suppresses the transcriptional activity of many human genes, ultimately affecting various biological processes that contribute to cancer progression [7, 10, 12, 21]. HMGA2 promotes entry into the S phase of the cell cycle by increasing E2F1 activity [8, 11] and inhibits apoptosis by stimulating the PI3K/Akt pathway, reducing p53 expression, and increasing the expression of the anti-apoptotic protein BCL2 [24, 25]. HMGA2 also plays a role in stimulating angiogenesis and EMT by activating signal transduction pathways, creating a positive feedback loop that increases HMGA2 expression (e.g., TGF-β, MAPK, and PI3K pathways) [26, 27], which in turn decreases epithelial protein expression, such as E-cadherin, and increases mesenchymal protein expression, such as fibronectin and Snail1/2 [13]. This process results in the loss of cell polarization and adhesion, transforming epithelial-like cells into mesenchymal-like cells with invasive and migratory properties [26, 28–30].

Our study did not show any association between HMGA2 protein tissue levels and gender or smoking habits. This can be explained by the fact that protein tissue levels are related to the tumor’s histological properties, independent of physiological factors specific to sex and smoking habits.

Based on these findings, the detection of HMGA2 in tissue samples can be used as a supporting diagnostic marker for bladder cancer, aiding in the determination of its stage, grade, and muscle invasion status. This study had some limitations, including the relatively small number of patients, lack of follow-up after treatment, and the fact that it was not a multicenter study.

## Conclusion

The present study demonstrated that HMGA2 protein is overexpressed in bladder cancer tissues compared to adjacent normal tissues, with levels positively correlated with tumor stage, grade, and muscle invasion. Consequently, HMGA2 protein can be considered a possible marker for the incidence and progression of bladder cancer. However, further studies involving larger patient cohorts with various types and stages of bladder cancer are needed to confirm the significance of HMGA2 protein levels in the diagnostic approach for bladder cancer.

## Data Availability

All materials and all data generated during this study are included in this article.

## Abbreviations

Akt: Protein kinase B
BCL2: B-cell lymphoma 2
BMREC: Biomedical Research Ethics Board
DAB: DiAminoBenzidine
EMT: Epithelial-mesenchymal transformation
HMG: High Mobility Group
HRP: Horseradish peroxidase
IHC: ImmunoHistoChemical staining
MAPK: mitogen-activated protein kinase
MIBC: Muscle-Invasive Bladder Cancer
NMIBC: Non-Muscle Invasive Bladder Cancer
PI3K: Phosphatidylinositide 3-kinase
qRT-PCR: Quantitative revers transcription-polymerase chain Reaction
SPSS: Statistical Package for the Social Sciences
TGF-β: Transforming growth factor beta
TURBT: TransUrethral Resection of the Bladder Tumor
UTR: UnTranslated Region
WHO: World Health Organization
